# In Vivo Diuretic Efficacy, Antioxidant Capacity, and Mineral Profiling of Tree Gum Exudates from Sour Cherry (*Prunus cerasus*), Mahaleb (*Prunus mahaleb*), and Cherry (*Prunus avium*)

**DOI:** 10.3390/ph19091484

**Published:** 2026-09-17

**Authors:** Sadık Küçükgünay, Fatma Ergün, Aynur Kırbaş, Demirel Ergün

**Affiliations:** 1 Department of Medical Pharmacology, Faculty of Medicine, Kırsehir Ahi Evran University, 40100 Kırsehir, Türkiye; 2 Department of Nutrition and Dietetics, Faculty of Health Sciences, Kırsehir Ahi Evran University, 40100 Kırsehir, Türkiye; fatma.ergun@ahievran.edu.tr; 3 Department of Medical Biochemistry, Faculty of Medicine, Kırsehir Ahi Evran University, 40100 Kırsehir, Türkiye; aynur.kirbas@ahievran.edu.tr; 4 Experimental Animals Unit, Faculty of Medicine, Kırsehir Ahi Evran University, 40100 Kırsehir, Türkiye; demirel.ergun@ahievran.edu.tr

**Keywords:** *Prunus cerasus* tree gum exudate, *Prunus mahaleb* tree gum exudate, *Prunus avium* tree gum exudate, antioxidant effect, diuretic effect, natriuretic activity, saluretic index

## Abstract

**Background and Objectives:** In this investigation, the mineral profile, antioxidant properties, and phenolic composition of tree gum exudates harvested from sour cherry (*Prunus cerasus*), mahaleb (*Prunus mahaleb*), and cherry (*Prunus avium*) were assessed, together with their in vivo diuretic efficacy in a Wistar albino rat model. **Materials and Methods:** Spectrophotometric quantification of total flavonoids and phenolics relied on aluminum nitrate and Folin–Ciocalteu reagents, respectively. Antioxidant potential was evaluated through radical scavenging tests, copper reduction capacity, and ferric reducing power, whereas ICP-MS was employed for elemental profiling. Rats were assigned to five groups: Control (distilled water), Furosemide (standard drug), PCRWE (group treated with *P. cerasus* tree gum exudate), PMRWE (group treated with *P. mahaleb* tree gum exudate), and PARWE (group treated with *P. avium* tree gum exudate). Urine samples collected at 5 and 24 h post administration were assessed for volume and Na^+^, K^+^, and Cl^−^ electrolyte concentrations to evaluate natriuretic and carbonic anhydrase activities. **Results:** Tree gum exudates contained rich mineral profiles, high phenolic and flavonoid levels, and strong antioxidant capacities. Cherry and mahaleb tree gum exudates showed low short-term and moderate long-term diuretic activity. In contrast, sour cherry tree gum exudate exhibited moderate short-term and high long-term diuretic activity. Furthermore, tree gum exudates significantly enhanced sodium, and potassium ion excretion compared to furosemide, demonstrating notable natriuretic and carbonic anhydrase activities. **Conclusions:** Tree gum exudates represent promising bioactive ingredients, antioxidants, or natural preservatives for food products. Specifically, sour cherry tree gum exudate displays significant diuretic and natriuretic potential, positioning it as a promising natural therapeutic agent for fluid balance management. Further research is required to isolate the active compounds and elucidate their specific mechanism on renal function.

## 1. Introduction

Diuretics work by acting through different anatomical regions and mechanisms in the kidneys (loop of Henle, distal tubule, carbonic anhydrase, aldosterone receptors, or Na^+^ channels) to eliminate excess fluid from the body [1]. Although primarily used in the treatment of hypertension, edema, heart and kidney failure [2], they have side effects such as hypovolemia, electrolyte imbalances, and lipid profile changes [3]. Their use before exercise can lead to fatigue and cramps [4]. The negative effects of these synthetic agents, which carry a risk of abuse and require lifestyle changes, have led researchers to explore herbal sources, significantly increasing studies on natural diuretic alternatives in recent years [5,6]. Plants rich in phytochemical constituents have been widely used as diuretics throughout history [7]. The literature reports that plant-based constituents can offer greater efficacy and a superior clinical profile compared to synthetic drugs [8]. This therapeutic potential and the potent diuretic properties of plants have also been confirmed by numerous scientific studies spanning from the past to the present [9].

Sour cherry (*P. cerasus*), mahaleb (*P. mahaleb*), and cherry (*P. avium*) are cultivated in many countries around the world for their fruits and seeds. Besides their nutritional properties, the fruits, seeds, and other products derived from these plants have uses in health, furniture, and cosmetics. In particular, the leaves and flowers of *P. cerasoides* are known to be used in the treatment of kidney stones and sand, while the trunk of the *P. cerasus* tree is known to be beneficial for some heart conditions, pitta imbalances, and skin discoloration [10]. Different tissues of *P. mahaleb* have long been utilized in ethnomedicine to manage conditions such as diabetes, digestive disorders, and several other health issues. Additionally, seeds of mahaleb, which find broad application across the food sector, are noted for their therapeutic role in mitigating pediatric diarrhea [11]. To defend against external stressors, these trees exude substantial quantities of trunk gum [12]. Appearing as drop-shaped or irregular masses, these exudates are originally pliable with a hue ranging from off-white to light yellow, eventually solidifying and turning darker over time. The exudation process peaks during warm summer months whereas it decelerates significantly in wintertime. Referred to as gums or resins, these edible substances are non-toxic, possess a bittersweet flavor profile, and are traditionally considered to exert diuretic activity. Moreover, they are recognized for boosting immune responses as well as managing serum glucose and lipid concentrations [13,14].

The objective of the present investigation was to evaluate the bioactive metabolite composition, antioxidant status, and elemental profile of tree gum exudates from three *Prunus* species—sour cherry (*P. cerasus*), mahaleb (*P. mahaleb*), and cherry (*P. avium*)—harvested within a shared geographical area. Furthermore, the study sought to validate their reputed diuretic efficacy in vivo employing a Wistar albino rat experimental model.

## 2. Results

In this study, the TPC [F(2,12) = 1143.916, *p* = 0.001], TFC: [F(2,12) = 114.789, *p* = 0.001], CUPRAC [F(3,16) = 1432.58, *p* = 0.001], FRAP [F(3,16) = 8469.033, *p* = 0.001] and IC_50_ [F(3,16) = 4019.378, *p* = 0.001] values of tree gum exudates were determined (Table 1). Differences between groups were considered significant at the *p* < 0.05 level.

Using ICP-MS analysis, trace elements (Mn and Cr), microelements (Zn and Cu), and macroelements (P, Fe, Na, Mg, K, and Ca) were evaluated in the tree gum exudate samples and the results are given in Table 2.

In the current research, urine volumes between the groups were evaluated. Significant differences were found at 5 h [F(4,20) = 26.481 *p* = 0.001] and 24 h [F(4,20) = 12.080, *p* = 0.001] (Table 3). Evaluation of the diuretic efficacy of tree gum exudate extracts relative to the control cohort revealed that furosemide treatment elicited the most pronounced response at both 5 h [F(4,20) = 17.314 *p* = 0.009] and 24 h [F(4,20) = 14.143, *p* = 0.001]. In addition, in the study in which the diuretic activity against furosemide was determined, the highest activity was found in the PCRWE group at the end of 5 h [F(4,20) = 30.425 *p* = 0.001] and 24 h [F(4,20) = 21.700, *p* = 0.001] (Table 3).

In this study, the concentrations of Na^+^ [F(4,20) = 13.148, *p* = 0.001], K^+^ [F(4,20) = 17.526, *p* = 0.001], Cl^−^ [F(4,20) = 27.682, *p* = 0.001], Ca^++^ [F(4,20) = 16.626, *p* = 0.015], Mg^++^ [F(4,20) = 23.542, *p* = 0.001], creatinine [F(4,20) = 23.026, *p* = 0.001] and uric acid [F(4,20) = 2.721, *p* = 0.059] were measured in the urine samples collected at the end of 24 h (Table 4).

In this study, the saluretic index [Na^+^: F(4,20) = 5.298, *p* = 0.004, K^+^: F(4,20) = 3.965 *p* = 0.016, Cl^−^: F(4,20) = 2.052, *p* = 0.126,], natriuretic activity [F(4,20) = 1.530, *p* = 0.232] and ion quotient [F(4,20) = 0.700 *p* = 0.601] values of the application groups were determined after 24 h (Table 5).

## 3. Discussion

Worldwide, the incidence of hypertension and cardiovascular diseases is rapidly increasing due to sedentary lifestyles and obesity. The therapeutic utility of diuretics has thus been further solidified, highlighting their crucial role as a first-line therapy for both heart failure with symptoms and hypertension. The search for improvement in existing treatment options and the discovery of new alternatives has popularized interest in herbal studies with diuretic effects. Phenolic compounds found in plants are known to trigger diuretic activity by playing a role in kidney function [15]. In the present study, the cherry tree gum exudate exhibited the highest total phenolic (120.15 ± 1.40 mgGAE/g) and flavonoid contents (62.28 ± 5.45 mgQE/g). Furthermore, mineral analyses of the tree gum exudate samples were performed, and it was found that they contained significant levels of minerals. The minerals found in sour cherry tree gum exudate followed the pattern of Cu < Cr < Mn < Zn < Fe < Na < P < K < Mg < Ca; in mahaleb tree gum exudate, the pattern of Cu < Cr < Zn < Mn < Na < Fe < P < Mg < K< Ca; and in cherry tree gum exudate, the pattern of Cu < Cr < Zn < Mn < Fe < Na < P < Mg < K < Ca. Specifically, sour cherry tree gum exudate showed the greatest levels of Na, Cr, Fe, and Zn, mahaleb tree gum exudate was richest in Mg, K, Ca, Mn, and Cu, while P reached its peak concentration in cherry tree gum exudate. These results are consistent with the literature [16,17,18]. In this study, differences in mineral concentrations of tree gum exudates affected diuretic activity. It is known that there is a direct correlation between diuretic effect and dietary Na intake, and an inverse correlation between diuretic effect and K intake [19]. The mineral structure of sour cherry tree gum exudate, which showed the highest diuretic activity in this study (high Na content, low K content), supports this.

In metabolism, an increase in extracellular fluid sodium concentration leads to an increase in extracellular fluid volume, which affects urine volume. To better observe the differences in diuretic effect and activity in this study by increasing urine volume, all rats fed an 18 h diet were given a fluid load of 0.9% NaCl [20,21]. Differences in urine volume between groups varied over time. Urine volumes measured in the tree gum exudate-treated groups were higher than the control group. Compared to the furosemide-treated group, urine volumes were lower at the end of 5 h, but similar at the end of 24 h. Similar differences between the tree gum exudate-treated groups persisted until the end of 24 h, with the highest urine volume observed in the sour cherry tree gum exudate-treated group. The diuretic effects of the gum-treated groups were calculated by comparing them to the control group. As a result, it was determined that the groups treated with tree gum exudate had good diuretic effects, higher than the control group, and lower than the group treated with furosemide. Among the groups treated with tree gum exudate, the group treated with sour cherry tree gum exudate showed the highest diuretic effect at 5 and 24 h. To better understand the diuretic effect, the study also calculated the diuretic activities of the treatment groups against furosemide. Taking furosemide as a reference value of 1.0, maximum diuretic action within the experimental groups was recorded for sour cherry tree gum exudate (0.75 after 5 h and 1.05 after 24 h). Diuretic activity is considered ineffective if less than 0.50, low if between 0.50 and 0.69, moderate if between 0.70 and 0.89, and high if equal to or greater than 0.90 [22,23]. When the results of our study were evaluated, it was determined that cherry and mahaleb tree gum exudates had low diuretic activation in the short term and moderate diuretic activation in the long term, while sour cherry tree gum exudate had moderate diuretic activity in the short term and high diuretic activity in the long term.

The high diuretic effect and activity we found in the application groups in this study may be due to the high amounts of phenols and flavonoids in the tree gum exudates. This is because polyphenols, and especially flavonoids, found in the structure of plants are known to have a diuretic effect [24]. This effect is mostly due to flavonoids being natural antagonists of adenosine receptors. The diuretic effect resulting from this interaction is shaped by vasodilation in the renal arteries [25]. In addition, diuretic activity is associated with the inhibition of angiotensin converting enzyme, an important protein that controls fluid balance and blood pressure in the body, the release of endothelial nitric oxide which increases glomerular filtration rate and reduces salt and water reabsorption, and the regulation of Prostaglandin E2 [26]. Oxidative stress in the body causes an increase in reactive oxygen species in the renal medulla, which in turn reduces endothelial nitric oxide release. Conversely, a diuretic action is facilitated through the enhancement of endothelial nitric oxide levels by bioactive flavonoids and phenolics exhibiting antioxidant properties [27]. The results we found in this study support what is stated in the literature.

The renal elimination of minerals such as chloride, potassium, and sodium through the kidneys plays a significant role in the development of heart failure, kidney failure, and high blood pressure [28]. Therefore, the excretion rates of these specific minerals hold equal therapeutic weight to overall fluid excretion in the treatment of these diseases. The amount of these minerals excreted in urine is considered an indicator of saluretic activity [29]. In our study, where the amount of minerals excreted in urine and saluretic activity were determined, Na^+^ excretion was found to be higher than K^+^ excretion in all groups. Furthermore, the urinary elimination of chloride, potassium, and sodium exceeded that observed in the furosemide group across all treatments. This outcome is likely driven by plasma mineral concentrations and a furosemide-like effect of tree gum exudates on Henle’s loop ascending segment. It was determined that the highest saluretic activity in terms of sodium and potassium excretion was in the group treated with mahaleb tree gum exudate, while the highest saluretic activity in terms of chloride excretion was in the group treated with cherry tree gum exudate. In addition, in order to better predict the diuretic mechanisms of tree gum exudates, their Na^+^/K^+^ ratio, i.e., natriuretic activities, were determined as biomarkers. In renal function profiling, a Na^+^/K^+^ quotient exceeding unity (>1.0) denotes effective diuretic activity without inducing excessive kaliuresis (potassium excretion). Ratios above 2.0 reflect a pronounced natriuretic response, whereas values exceeding 10.0 signify a distinct potassium-conserving mechanism [30]. In the present study, the calculated ratios fell within the range of 1.0 to 2.0 across all treatment groups. This finding confirms that the tested tree gum exudates promoted adequate diuresis while preserving potassium balance, highlighting their potential as viable diuretic agents. Furthermore, the urinary ion quotient (Cl^−^/(Na^+^ + K^+)^ serves as an indicator for carbonic anhydrase activity; values maintained between 0.8 and 1.0 suggest that carbonic anhydrase inhibition is clinically negligible, whereas lower ratios imply varying degrees of enzyme inhibition [29,30]. The ion quotient values obtained in this work consistently exceeded 0.8, demonstrating that the diuretic action of these exudates operates independently of carbonic anhydrase suppression.

## 4. Materials and Methods

### 4.1. Reagents and Chemical Materials

All chemicals, solvents, and reference reagents utilized in the study were of analytical purity grade and purchased from commercial suppliers. Sodium chloride was obtained from Poliflex^®^ (İstanbul, Türkiye). Standards and indicators including gallic acid, quercetin, Folin–Ciocalteu reagent, butylated hydroxytoluene, 1,1-diphenyl-2-picrylhydrazyl were acquired from Merck^®^ (Merck KGaA, Darmstadt, Germany). Additionally, furosemide (Lasix^®^, Sanofi, Istanbul, Türkiye) was provided for comparison, whereas high-purity methanol and ethanol were procured from Sigma-Aldrich^®^ (Merck KGaA, Darmstadt, Germany).

### 4.2. Devices and Materials

Standard laboratory equipment was used in the study, including metabolic cages (TECNIPLAST^®^,Buguggiate, Italy), oral gavages (22 gauge; Intech, Shenzhen, China), digital scale (Abron Exports, Ambala Cantt, India), rotary evaporator (Yamato Scientific Co. Ltd., Tokyo, Japan), optical liquid concentration meter (HANNA^®^ Nușfalău, Romania), digital pH meter (MW150 MAX, Milwaukee Instruments, Cluj-Napoca, Romania), and Ion Selective Electrode analyzer (Alinity (Abbutt, Abbott Park, IL, USA)) and Induced Coupled Plasma Mass Spectrometer (Agilent Technologies, Santa Clara, CA, USA). In addition, basic laboratory supplies such as beakers, Erlenmeyer flasks, and Whatman No. 1 filter paper were used for various experimental procedures.

### 4.3. Tree Gum Exudate Collection

The tree gum exudates from the *P. cerasus*, *P. mahaleb*, and *P. avium* fruit trees used in this study were collected in July-August 2025 from the farm located in Olur district of Erzurum region of Türkiye (40°49′49.28″ N 42°07″55.06 E. 1317 m, 40°49′50.73″ N 42°07″56.91 E. 1315 m, 40°49′47.97″ N 42°07″54.89 E. 1319 m). Previous similar studies played a role in determining July as the harvest season [16,31]. Field experts at the facility identified the trees targeted for tree gum exudate collection. To create representative composite samples for the study, tree gum exudate was gathered from a minimum of three trees per group and thoroughly mixed. Voucher specimens for a portion of the collected tree gum exudate were subsequently archived in the herbarium at the Kırsehir Ahi Evran University Faculty of Health Sciences Laboratory (DE-1001, DE-1002, DE-1003).

### 4.4. Preparation and Analysis of Tree Gum Exudate Extracts

The tree gum exudate samples were washed and dried in the shade. The dried samples were powdered using a mechanical grinder. A portion of each tree gum exudate sample was placed into erlenmeyer flasks. Methanol, in an amount equal to 20 times their mass, was added to them. The mixtures were stirred overnight using a magnetic stirrer. The obtained extracts were filtered using Whatman No. 1 filter paper. Subsequently, methanol was removed from the dry extracts in a rotary evaporator at 45 °C. Finally, the concentrated tree gum exudate extracts were used for phytochemical analysis, mineral content analysis, and testing of diuretic activity.

### 4.5. Chemical Analyses of Tree Gum Exudates3

#### 4.5.1. Quantification of Total Phenolic Content

The total phenolic content (TPC) of the tree gum exudate extracts was evaluated using a colorimetric method with Folin–Ciocalteu reagent (FCR) [32]. In brief, an aliquot of 100 μL from each extract stock (1000 ppm dissolved in methanol) was transferred into reaction tubes, mixed with 100 μL of FCR. It was then diluted with 4.5 mL of deionized water. Following a 3 min standing period at room temperature, 300 μL of a 2% (*w*/*v*) sodium carbonate (Na_2_CO_3_) solution was introduced, and the resulting mixture was homogenized by vortexing. The solution was subsequently allowed to react in the dark for 2 h at ambient conditions. Absorbance values were recorded at 760 nm employing a UV-Vis spectrophotometer. TPC levels were derived using a linear regression curve generated from gallic acid reference standards, with values reported as milligrams of gallic acid equivalents per gram of dry extract (mg GAE/g).

#### 4.5.2. Determination of Total Flavonoid Content

The total flavonoid content (TFC) of the tree gum exudate extracts was quantified employing the aluminum nitrate Al(NO_3_)_3_ colorimetric assay adapted from Moreno et al. [33]. Briefly, a 0.5 mL sample of each extract stock (1000 ppm in methanol) was combined with 0.1 mL of 1 M potassium acetate (CH_3_COOK) in reaction tubes. Following a 1-min equilibration, 0.1 mL of 10% (w/v) Al(NO_3_)_3_ reagent was introduced, and the solution was thoroughly homogenized. The overall mixture volume was subsequently adjusted to 5 mL using ethanol. After incubating for 40 min at ambient conditions, optical density was measured at 415 nm via a UV-Vis spectrophotometer. TFC values were computed using a standard calibration plot generated with quercetin, with final concentration values reported as milligrams of quercetin equivalents per gram of dry extract (mg QE/g).

#### 4.5.3. Ferric Reducing Power Assay

The ferric reducing potential of the tree gum exudate extracts was determined following the methodology outlined by Oyaizu (1986) [34]. A reference calibration curve was established using Trolox standard solutions spanning a concentration range of 25–750 μg/mL. A 0.5 mL volume of each extract (1000 ppm ppm in methanol), reference antioxidant, or Trolox standard was combined with 2.5 mL of 0.2 M phosphate buffer (pH 6.6) and 2.5 mL of $1\%\;\left(w/v\right)$ potassium ferricyanide (K_3_[Fe(CN)_6_]). The resulting mixture was incubated in a water bath at 50 °C for 20 min. To terminate the reaction, 2.5 mL of 10% (*w*/*v*) trichloroacetic acid (TCA) was added to the tube. The solution was centrifuged (at 2000 rpm for 10 min). Then, 2.5 mL of the clear supernatant was taken and diluted with an equal volume of deionized water. Finally, 0.5 mL of 0.1% (*w*/*v*) FeCl_3_ was added. The absorbance of the reaction mixture was recorded at 700 nm using a UV-Vis spectrophotometer. Butylated hydroxytoluene (BHT) was assessed under identical experimental conditions as a positive control. All measurements were conducted in quintuplicate, with outcomes expressed as millimoles of Trolox equivalents per gram of dry extract (mmol TE/g).

#### 4.5.4. Cupric Ion Reducing Antioxidant Capacity (CUPRAC) Determination

The cupric ion reducing power of the exudate preparations was quantified in accordance with the protocol reported by Apak et al. (2004) [35]. A linear standard curve was generated utilizing Trolox solutions across a concentration gradient of 25–250 μg/mL. Briefly, 1.0 mL of copper(II) chloride (CuCl_2_), 1.0 mL of neocuproine solution, and 1.0 mL of ammonium acetate buffer were combined in a reaction tube. To this mixture, 0.5 mL of the respective Trolox standard was added, followed by the addition of 0.5 mL of deionized water to achieve the final volume. The reaction vessel was sealed and allowed to incubate at ambient temperature for 30 min. Subsequently, optical density was recorded at 450 nm against a reagent blank using a UV-Vis spectrophotometer. Identical procedural steps were performed for the tree gum exudate extracts (1000 ppm) as well as butylated hydroxytoluene (BHT), which served as the positive reference control. All experimental assays were executed in quintuplicate, with values expressed as milligrams of Trolox equivalents per gram of dry extract (mg TE/g).

#### 4.5.5. DPPH Radical Scavenging Capacity

The radical-quenching potential of the tree gum exudate extracts and positive control was measured via the 1,1-diphenyl-2-picrylhydrazyl (DPPH) free radical assay based on the procedure established by Blois (1958) [36]. A series of methanolic dilutions were prepared for each extract and standard compound spanning concentrations from 50 to 200 μg/mL. In brief, a 0.5 mL sample of each solution was combined with 2.5 mL of a freshly prepared 0.1 mM methanolic DPPH solution. The resulting mixture was vortexed. It was then left to stand in the dark at room temperature for 30 min. At the end of this period, the absorbance of the reaction mixture was measured against a methanol blank sample at 517 nm using a UV-Vis spectrophotometer. Butylated hydroxytoluene (BHT) was analyzed under parallel conditions as the positive reference compound. The radical scavenging activity—expressed as percentage inhibition (%*I*)—was derived using the following formula:

$$\mathrm{DPPH}\;\mathrm{Radical}\;\mathrm{Scavenging}\;\mathrm{Activity}\;(\%)=\left(\frac{A_{\mathrm{blank}}-A_{\mathrm{sample}}}{A_{\mathrm{blank}}}\right)\times 100$$

where $A_{\mathrm{blank}}$ is the absorbance of the control reaction (containing all reagents except the extract or standard) and $A_{\mathrm{sample}}$ is the absorbance of the test sample or standard. All assays were conducted in quintuplicate. To determine the concentration required to inhibit DPPH radicals by 50% (IC_50_), a graph was plotted showing the percentage of inhibition against the sample concentration. IC_50_ values were determined from the linear regression of the graph curves.

#### 4.5.6. Quantitative Elemental Profiling via ICP-MS

The elemental composition of the samples was evaluated using inductively coupled plasma mass spectrometry (ICP-MS, Agilent 7900) following microwave-assisted acid digestion. In brief, precisely weighed powdered samples (approximately 10–20 mg) were digested with 2 mL of concentrated nitric acid (${\mathrm{HNO}}_{3}$) and 3 mL of hydrogen peroxide ($H_{2}O_{2}$) in a closed-vessel microwave digestion unit (Milestone). Following the temperature- and pressure-monitored digestion procedure, the resulting clear solutions were completed to a final volume of 10 mL using ultrapure water. Multi-element calibration standard curves were constructed across a concentration range from 0 to 50 ppb (0, 1, 5, 10, 20, 30, 40, and 50 ppb). Prior to sample measurement, optimization and tuning of the instrument were performed using a standard tuning solution containing 200 ppb of Li, Yb, and Cs. To account for matrix effects and signal drift, an internal standard solution containing 200 ppb of scandium (Sc) and indium (In) was fed online into the system during analysis. Subsequently, the prepared samples were introduced into the ICP-MS system under optimized operating conditions [37].

### 4.6. Determination of Diuretic Activity

**Figure 1:**
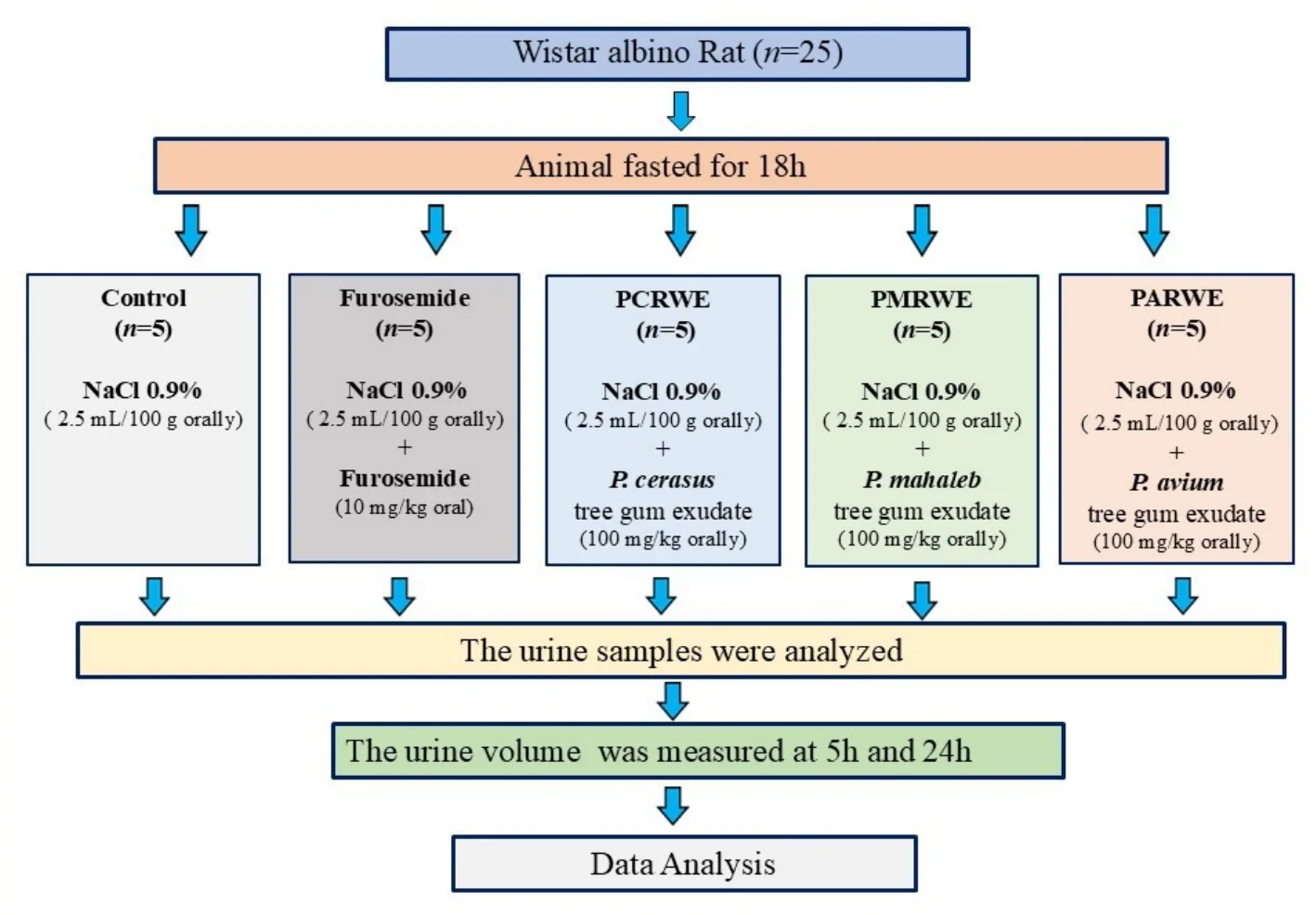
The flowchart of the experimental design.

The flowchart of the experimental design used to determine diuretic activity is given in Figure 1. All experimental procedures performed on animals in the study were approved by the Kırşehir Ahi Evran University Local Ethics Committee for Animal Experiments (10 June 2026, 11/3) and conducted in accordance with the ARRIVE guidelines. In this study, 25 healthy rats, 12 weeks old and weighing 200–210 g, born from the stock breeding herd raised at the Animal Experiments Unit of Kırşehir Ahi Evran University Faculty of Medicine, were used. Throughout the study, the rats were housed under an artificial 12 h light/12 h dark cycle (lights on from 7:00 to 19:00) at a constant temperature of 22 ± 2 °C and a relative humidity of 65%. Prior to the study, the animals were fed ad libitum with standard rat chow and water. Inclusion criteria for the animals were defined as being healthy, 12 weeks of age, and within the specified body weight range (200–210 g). No exclusion criteria were pre-set, and no animals was excluded from the study or the final analysis. No blinding was performed in this study; the investigators were aware of the group allocations during the experimental procedures, data collection, and outcome assessment. To estimate the appropriate sample size, a power analysis was executed via MINITAB statistical software (Minitab 17) based on the reference literature, establishing a group size of five animals per group (a total of 25 animals). The experimental design comprised five distinct groups (*n* = 5 per group): a negative control group, a reference/positive control group receiving Furosemide (10 mg/kg), and three experimental groups treated with 100 mg/kg of aqueous tree gum exudate extracts from *P. cerasus* (PCRWE), *P. mahaleb* (PMRWE), and *P. avium* (PARWE), respectively. Prior to urine harvesting, feed and fluid intake were restricted for an 18 h fasting window. Subsequently, a uniform physiological fluid and electrolyte challenge was induced across all subjects by administering isotonic saline solution (0.9% NaCl at 2.5 mL/100 g body weight) using an intragastric gavage needle (INTECH^®^ 22ga) [9,38]. Directly after saline loading, the reference drug or respective aqueous exudate preparations were orally delivered, ensuring all groups received an equivalent total oral fluid intake. Rats were individually housed in metabolic cages to measure excreted urine volumes at two distinct time points: 5 h (short period) and 24 h (long period). The resulting parameters were utilized to determine the diuretic effect and diuretic activity based on established mathematical formulas [39,40].


$$Diuretic\;effect=\frac{The\;average\;urine\;volume\;of\;the\;test\;group}{The\;average\;urine\;volume\;of\;the\;control\;group}$$

$$Diuretic\;activity=\frac{The\;diuretic\;action\;of\;test\;group}{The\;diuretic\;action\;of\;furosemide\;group}$$

### 4.7. Urinary Electrolyte and Biochemical Profiling

Urine specimens collected over the 24 h period were analyzed to quantify excreted electrolytes (Na^+^, K^+^, and Cl^−^) along with creatinine, uric acid, and urea contents using an automated Ion-Selective Electrode system (Alinity, Abbott, Abbott Park, IL, USA). Ratios of Na^+^/K^+^ and Cl^−^/K^+^ + Na^+^, alongside saluretic indices, were calculated to estimate natriuretic capacity and possible carbonic anhydrase inhibitory effects. Furthermore, ion quotient parameters and natriuretic activity scores were computed according to previously reported protocols [22].


$$Natriuretic\;activity=\frac{\left[Na\;\right]}{\left[K\;\right]}$$

$$Ion\;quotient=\frac{\left[Cl\right]}{\left[Na+K\;\right]}$$

### 4.8. Statistical Analyses

All numerical data were processed statistically using IBM SPSS Statistics software (Version 22.0). The Shapiro–Wilk test was applied to check for normal distribution, while equality of variances was assessed through Levene’s test. One-way ANOVA was applied to data showing a normal distribution. Where statistically significant variation was established, Duncan’s multiple range post hoc test was applied to pinpoint specific pairwise group distinctions [41]. All values were considered significant at *p* < 0.05.

## 5. Conclusions

In this study, no pathological conditions were observed in rats given tree gum exudates. Furthermore, it was observed that the tree gum exudates used in the study had no dose-dependent adverse physiological or developmental effects on the rats. The absence of any clinical stress or abnormality during this study provides evidence that the tree gum exudates did not induce acute inflammation or oxidative stress in the kidney tissue. The data obtained support the traditional claim that the tree gum exudates used in the study have a diuretic effect. The findings show that tree gum exudates at a dosage of 100 mg/kg display varying degrees of ion quotient, natriuretic, saluretic, and diuretic activities depending on the type of fruit from which they originate. Hence, the results suggest that sour cherry, mahaleb, and cherry tree gum exudates represent viable candidates for diuretic use. Furthermore, additional investigations ought to be carried out to evaluate the renal and antihypertensive impacts of these tree gum exudates, whose diuretic potential has been established here. Nevertheless, the results obtained in this study are promising. From a clinical perspective, these findings indicate that the extract in question could serve as a supportive natural agent in managing fluid retention (edema) and mild-to-moderate hypertension in humans. In translating these results to human physiology, differences in metabolic rate and body surface area must be taken into account to ensure health preservation and satisfy regulatory requirements. However, human clinical trials on both healthy volunteers and patients are strictly required to evaluate the precise human equivalent doses, pharmacokinetics, and long-term safety profile of the extract.

## Figures and Tables

**Table 1:** Phytochemical profile and antioxidant properties of studied tree gum exudates.

	TPC (mg GAE/g)	TFC (mg QE/g)	CUPRAC (mg TE/g)	FRAP (mmol TE/g)	IC_50_ (µg/mL)
*P. cerasus* tree gum exudate	61.00 ± 3.87 ^b^	41.18 ± 0.65 ^b^	443.60 ± 16.86 ^b^	0.87 ± 0.01 ^c^	136.00 ± 3.08 ^b^
*P. mahaleb* tree gum exudate	47.05 ± 1.66 ^c^	31.53 ± 1.44 ^c^	147.91 ± 7.21 ^d^	0.67 ± 0.01 ^d^	198.00 ± 1.87 ^a^
*P. avium* tree gum exudate	120.15 ± 1.40 ^a^	62.28 ± 5.45 ^a^	345.95 ± 12.38 ^c^	1.53 ± 0.03 ^b^	60.80 ± 0.83 ^d^
BHT	-	-	1167.00 ± 47.67 ^a^	3.54 ± 0.04 ^a^	105.60 ± 1.67 ^c^

The group means, represented by similar letter codes (a, b, c, d) in the table, exhibit an equivalent and homogeneous distribution with respect to the respective parameter (*p* < 0.05). TPC, Total phenolic content; TFC, total flavonoid content; FRAP, Fe^3+^-Fe^2+^ reducing power; CUPRAC, copper (II) reducing capacity; GAE, gallic acid equivalent; QE, quercetin equivalent; TE, trolox equivalent; IC_50_, Concentration that inhibits DPPH by 50%; BHT, Butylated Hydroxytoluene.

**Table 2:** ICP-MS analysis table of tree gum exudate samples (ppm).

	Na	Mg	P	K	Ca	Cr	Mn	Fe	Cu	Zn
*P. cerasus* tree gum exudate	147.34	1467.00	1172.83	1406.02	3473.60	1.86	28.17	135.08	0.42	37.14
*P. mahaleb* tree gum exudate	124.91	2028.75	1009.71	2145.02	3856.22	1.08	31.73	134.50	1.02	2.74
*P. avium* tree gum exudate	134.34	1305.78	1207.77	2066.35	2469.60	1.28	23.24	110.74	0.54	4.21

Ca: Calcium; Cu: Copper; Cr: Chromium; Fe: Iron; K: Potassium; Mg: Magnesium; Mn: Manganese; Na: Sodium; P: Phosphorus; Zn: Zinc.

**Table 3:** Results of urine volume, diuretic effect, and diuretic activity.

	5 h			24 h		
Groups	V (mL/100 g)	DE	DA	V (mL/100 g)	DE	DA
Control	1.23 ± 0.12 ^c^	1 ^c^	0.49 ± 0.04 ^c^	1.63 ± 0.15 ^c^	1 ^c^	0.50 ± 0.09 ^d^
Furosemide	2.46 ± 0.32 ^a^	2.10 ± 0.23 ^a^	1 ^a^	3.10 ± 0.21 ^a^	2.00 ± 0.21 ^a^	1 ^ab^
PCRWE	1.78 ± 0.39 ^b^	1.47 ± 0.49 ^b^	0.75 ± 0.17 ^b^	2.93 ± 0.54 ^a^	1.90 ± 0.34 ^ab^	1.05 ± 0.15 ^a^
PMRWE	1.15 ± 0.23 ^c^	0.92 ± 0,12 ^c^	0.50 ± 0.04 ^c^	2.29 ± 0.47 ^b^	1.64 ± 0.43 ^b^	0.72 ± 0.11 ^c^
PARWE	1.32 ± 0.06 ^c^	1.07 ± 0.15 ^c^	0.53 ± 0.06 ^c^	2.76 ± 0.32 ^ab^	1.82 ± 0.22 ^ab^	0.89 ± 0.10 ^b^

Control, distilled water; Furosemide, group given a 10 mg/kg dose of furosemide; PCRWE, group given a 100 mg/kg dose of *P. cerasus* tree gum exudate; PMRWE, group given a 100 mg/kg dose of *P. mahaleb* tree gum exudate; PARWE, group given a 100 mg/kg dose of *P. avium* L tree gum exudate; V, Volume; DE, Diuretic effect; DA, Diuretic activity. The group means, represented by similar letter codes (a, b, c, d) in the table, exhibit an equivalent and homogeneous distribution with respect to the respective parameter (*p* < 0.05).

**Table 4:** Urine electrolyte content.

	Urinary Electrolyte Concentration						
	Na^+^ (mmol/L)	K^+^ (mmol/L)	Cl^−^ (mmol/L)	Ca^++^ (mg/dL)	Mg^++^ (mg/dL)	Creatinine (mg/dL)	Uric Acid (mg/dL)
Control	200.40 ± 23.35 ^a^	163.80 ± 13.14 ^a^	196.40 ± 19.80 ^b^	5.68 ± 1.20 ^ab^	13.60 ± 1.14 ^b^	192.00 ± 16.49 ^a^	3.38 ± 0.93 ^a^
Furosemide	152.00 ± 2.51 ^b^	121.20 ± 4.32 ^d^	170.20 ± 12.27 ^c^	7.80 ± 2.53 ^a^	16.21 ± 0.70 ^a^	78.80 ± 6.68 ^b^	5.44 ± 0.25 ^a^
PCRWE	200.20 ± 23.35 ^a^	144.26 ± 8.97 ^c^	139.90 ± 10.29 ^d^	4.56 ± 1.34 ^b^	8.80 ± 1.34 ^c^	101.40 ± 17.98 ^b^	4.04 ± 2.25 ^a^
PMRWE	197.20 ± 12.47 ^a^	156.20 ± 5.66 ^ab^	223.40 ± 15.22 ^a^	4.28 ± 0.52 ^b^	12.80 ± 1.30 ^b^	178.00 ± 43.20 ^a^	3.18 ± 1.04 ^a^
PARWE	208.40 ± 10.16 ^a^	146.46 ± 8.16 ^bc^	206.20 ± 9.31 ^ab^	4.56 ± 1.73 ^b^	17.02 ± 2.38 ^a^	172.20 ± 16.00 ^a^	3.24 ± 1.08 ^a^

Control, distilled water; Furosemide, group given a 10 mg/kg dose of furosemide; PCRWE, group given a 100 mg/kg dose of *P. cerasus* tree gum exudate; PMRWE, group given a 100 mg/kg dose of *P. mahaleb* tree gum exudate; PARWE, group given a 100 mg/kg dose of *P. avium* L tree gum exudate. The group means, represented by similar letter codes (a, b, c, d) in the table, exhibit an equivalent and homogeneous distribution with respect to the respective parameter (*p* < 0.05).

**Table 5:** Saluretic indices, natriuretic activity, and ion quotients of rats treated with tree gum exudates.

	Saluretic İndex		Natrıuretic Activity		Ion Quotient
	Na^+^	K^+^	Cl^−^	Na^+^/K^+^	Cl/(Na^+^ + K^+^)
Control	1 ^b^	1 ^a^	1 ^a^	1.40 ± 0.20 ^a^	1.70 ± 0.0 ^a^
Furosemide	0.78 ± 0.05 ^c^	0.76 ± 0.09 ^b^	0.81 ± 0.19 ^a^	1.21 ± 0.15 ^a^	0.93 ± 0.15 ^a^
PCRWE	0.89 ± 0.25 ^bc^	0.91 ± 0.14 ^a^	0.87 ± 0.23 ^a^	1.40 ± 0.27 ^a^	0.99 ± 0.21 ^a^
PMRWE	1.19 ± 0.17 ^a^	0.94 ± 0.07 ^a^	1.08 ± 0.19 ^a^	1.56 ± 0.30 ^a^	0.94 ± 0.09 ^a^
PARWE	0.94 ± 0.10 ^bc^	0.90 ± 0.10 ^a^	1.09 ± 0.12 ^a^	1.33 ± 0.19 ^a^	0.94 ± 0.17 ^a^

Control, distilled water; Furosemide, group given a 10 mg/kg dose of furosemide; PCRWE, group given a 100 mg/kg dose of *P. cerasus* tree gum exudate; PMRWE, group given a 100 mg/kg dose of *P. mahaleb* tree gum exudate; PARWE, group given a 100 mg/kg dose of *P. avium* tree gum exudate. The group means, represented by similar letter codes (a, b, c) in the table, exhibit an equivalent and homogeneous distribution with respect to the respective parameter (*p* < 0.05).

## Data Availability

The original contributions presented in this study are included in the article. Further inquiries can be directed to the corresponding authors.

## Abbreviations

The following abbreviations are used in this manuscript:

BHT	2,6-di-t-butyl-1-hydroxytoluene
Ca	Calcium
Cr	Chromium
Cu	Copper
CUPRAC	Copper II reducing capacity
DA	Diuretic activity
DE	Diuretic effect
DPPH	1,1-diphenyl-2-picrylhydrazyl
Fe	Iron
FCR	Folin–Ciocalteu reagent
FRAP	Fe^3+^-Fe^2+^ reducing power
GAE	Gallic acid equivalent
IC_50_	Concentration that inhibits DPPH by 50%.
ICP-MS	Inductively coupled plasma mass spectrometry
K	Potassium
Mg	Magnesium
Mn	Manganese
Na	Sodium
NaCl	Sodium chloride
P	Phosphorus
PARWE	Group given a 100 mg/kg dose of *P. avium* tree gum exudate
PCRWE	Group given a 100 mg/kg dose of *P. cerasus* tree gum exudate
PMRWE	Group given a 100 mg/kg dose of *P. mahaleb* tree gum exudate
QE	Quercetin equivalent
TE	Trolox equivalent
TFC	Total flavonoid content
TPC	Total phenolic content
V	Volume
Zn	Zinc
